# Decolorization and Detoxification of Synthetic Dyes by Mexican Strains of *Trametes* sp.

**DOI:** 10.3390/ijerph16234610

**Published:** 2019-11-20

**Authors:** Laura N. Levin, Carlos E. Hernández-Luna, Guillermo Niño-Medina, Juan Pablo García-Rodríguez, Iosvany López-Sadin, Gerardo Méndez-Zamora, Guadalupe Gutiérrez-Soto

**Affiliations:** 1 Laboratorio de Micología Experimental, Departamento de Biodiversidad y Biología Experimental, Facultad de Ciencias Exactas y Naturales, Universidad de Buenos Aires, INMIBO-CONICET, Buenos Aires 1428 CABA, Argentina; lale@bg.fcen.uba.ar; 2 Laboratorio de Enzimología, Facultad de Ciencias Biológicas, Universidad Autónoma de Nuevo León, Pedro de Alba S/N., Ciudad Universitaria, San Nicolás de los Garza, Nuevo León C.P. 66455, Mexico; carlosehlmx@yahoo.com; 3 Universidad Autónoma de Nuevo León, Facultad de Agronomía, Francisco Villa S/N., Col. Ex Hacienda El Canadá, General Escobedo, Nuevo León C.P. 66050, Mexico; nino.medina.g@gmail.com (G.N.-M.); jpablo.garciar14@gmail.com (J.P.G.-R.); iosvanyls@gmail.com (I.L.-S.); mezage@hotmail.com (G.M.-Z.); 4 Departamento de Mecánica Aplicada, Facultad de Ciencias Técnicas, Universidad de Ciego de Ávila, Carretera a Morón, Km 9 1/2, Ciego de Ávila C.P. 69450, Cuba

**Keywords:** detoxification, dye decolorization, isoforms, laccase, waste water

## Abstract

Laccases have attracted a great deal of interest because of their remarkable ability for the degradation of synthetic dyes present in wastewaters. New laccase producing sources with robust operational and functional properties are being continuously explored. In this work, the potential for the decolorization and detoxification of synthetic dyes was evaluated in two Mexican strains of the genus *Trametes*. The decolorization capacity of *Trametes maxima* LE130 and *Trametes* sp. LA1 was tested in solid and liquid media. The phytotoxicity of the degradation products was determined using *Raphanus sativus* and *Pisum sativum* seeds. In solid media, both strains showed a higher decolorization capacity (*p* ≤ 0.05) than *Phanerochaete chrysosporium* ATCC 24725, which is known to be very efficient in lignin and dye-degradation. They produced laccase as the main ligninolytic enzyme; *T. maxima* LE130 secreted a single isoform of 43.9 kDa, while *Trametes* sp. LA1 produced three isoforms of 67.3, 58.6 and 52.7 kDa, respectively. *Trametes* sp. LA1 culture fluids were capable of decolorizing and detoxifying chemically diverse dyes (anthraquinonic dye Remazol Brilliant Blue R, azoic Reactive Black 5 and triphenylmethane Crystal Violet) without the addition of redox mediators. Therefore, this could be considered as a new laccase source which could be potentially competitive in the bioremediation of dye-containing wastewaters.

## 1. Introduction

Synthetic dyes are used globally in numerous industries, such as in the textile, paper printing, food, pharmaceutical, leather and cosmetics industries. They produce large amounts of colored liquid wastes containing toxic chemicals, some of which are non-biodegradable and carcinogenic and pose a major threat to the environment and human health [1]. Due to their synthetic origin and complex aromatic molecular structures, their treatment is often expensive and inefficient. To date, there is no highly effective technique which is capable of the complete removal of both the color and the toxic properties of the dyes released into the environment [1]. Microbial dye detoxification is promising because of its low-cost and relative ease of technological development. Nevertheless, azo compounds are susceptible of forming more hazardous products such as aromatic amines after being reduced or decolorized by bacterial azoreductases [2]. The role of fungi and their enzymes and their potential use in the degradation and detoxification of dyes has been well reported and recognized [3]. The most widely used of these are white rot fungi. Their nonspecific lignin-degrading systems—mainly composed of laccase, and peroxidases such as Mn-peroxidase (MnP) and lignin-peroxidase (LiP)—degrade dyes by oxidation [4,5]. The relative contribution of different enzymes in fungal cultures to decolorization is not yet completely understood [6]. As the enzymatic system secreted by basidiomycetes depends even on the strain and culture conditions, more white-rot fungi need to be screened for their ability to degrade dyes [7]. Laccases (benzenediol: oxygen oxidoreductases, EC 1.10.3.2) are considered ideal “green catalysts” as they do not require hydrogen peroxide for their oxidation reaction and use molecular oxygen as an electron acceptor, generating water. Among them, high-redox potential laccases, which may oxidize a wider range of substrates including those reported for peroxidases, are very attractive for biotechnological purposes [8]. A lower redox potential (E°) of the substrate and/or a higher E° of laccase normally results in a higher rate of substrate oxidation, and a linear correlation between the percentage of decolorization of each dye and the respective redox potential was found [9]. Recently, there has been growing interest in studying new fungal laccase sources, with the expectation of finding enzymes with novel properties or which are very robust for application in dye decolorization [10,11,12]. One of the limitations of the large-scale application of the enzyme is the lack of a capacity to produce large volumes of highly active enzyme. These problems can be solved with the use of recombinant organisms or with screening for natural hyper secretory strains [13]. The information available in the literature regarding the detoxification of textile effluents with laccases is scarce, and no strict correlation has been found between decolorization and detoxification [10,14,15,16]. The identification of the metabolites produced during the decolorization and/or biodegradation of a certain dye, as well as the evaluation of their toxicity, is an essential step for the eventual application of a laccase, assuring the safety of the treated effluents [3].

Mexico is considered to be one of the most mega-diverse countries of the world; it hosts more than 10% of the world’s biological diversity [17]. In Mexico, it is estimated that 200,000 fungal species occur, and only about 5% have been studied [18], indicating a lack of knowledge about this group of microorganisms and highlighting the necessity of performing further investigations. They represent an important resource from the technological point of view because they can be used in a broad variety of industrial processes. A screening for thermo-tolerant ligninolytic fungi with laccase, lipase and protease activity, isolated in the large area of forests known as the Huasteca Hidalguense in the state of Hidalgo (Mexico), was conducted by Cruz-Ramirez et al. [19]. Gutiérrez-Soto et al. [20] explored the lignocellulolytic potential of 74 strains isolated in northeastern Mexico. Several species of the genus *Trametes* (Aphyllophorales, Polyporaceae) are considered as good laccase producers (i.e., *Trametes versicolor*, *Trametes hirsuta* and *Trametes trogii*) [21]. In this work, the potential for the decolorization and detoxification of synthetic dyes was evaluated in two Mexican strains of the genus *Trametes*.

## 2. Materials and Methods

### 2.1. Fungal Strains and Culture Conditions

*Trametes maxima* LE130 and *Trametes* sp. LA1 were provided by the culture collection of the Laboratory of Enzymology, Biology Department from the UANL (Nuevo León Autonomous University). *Phanerochaete chrysosporium* ATCC 24,725 was used for comparison. All strains were conserved in YMGA (glucose 4 g L^−1^, malt extract 10 g L^−1^, yeast extract 4 g L^−1^ and agar 15 g L^−1^) slants at 4 °C with periodic subcultures every three months. The growth media consisted of liquid Bran Flakes medium (BF) (2% (w/v) Kellogg’s μ Bran Flakes) in 60 mM potassium phosphate buffer pH 6.0 [22] and the rich medium for Basidiomycota (BRM) reported by Bezalel et al. [23]. Additionally, the effects of MnSO_4_ (0.1 mM), FeSO_4_ (0.1 mM), CuSO_4_ (0.35 mM) and ethanol (3%) were evaluated as inducers of MnP, LiP and laccase activity, respectively [7,24,25]. Erlenmeyer flasks (500 mL) containing 200 mL of growth media were inoculated with 10 mm diameter plugs cut out from the margin of a 7-day-old colony growing on PDA media and incubated statically at 28 °C for 20 days. The final pH of all media was adjusted to 6.0. Cultures were harvested at proper intervals (every two days), and 2 mL aliquots of the supernatant were collected aseptically and used as enzyme sources. 

### 2.2. Enzymatic Assays

Laccase activity was determined by following the oxidation of 2,6 dimethoxyphenol (DMP) at 468 nm (ε_468_(molar extinction coefficient) = 49,600 M^−1^cm^−1^) [26]. MnP was measured at 270 nm (ε_270_ = 11,590 M^−1^cm^−1^) by the formation of Mn^3+^ malonate complexes according to Wariishi et al. [27]. LiP was measured with veratryl alcohol by following the formation of veratraldehyde at 310 nm (ε_310_ = 9300 M^−1^cm^−1^) [28]. Enzymatic activities were expressed in units (U) defined as the amount of enzyme required to produce 1 μmol of product. Enzymatic reactions were carried out at 25 °C and pH 3.5 and evaluated in a UV-Vis 1800 spectrophotometer (Shimadzu)).

### 2.3. Solid-Plate Dye Decolorization

All the strains were inoculated on agar plates in BF medium supplemented with 200 ppm of each of the dyes evaluated (depicted in Table 1). Inoculum consisted of a 50 mm agar disc of a 5 days old culture grown on YMGA. Non-inoculated plates served as controls for abiotic decolorization. Each fungus was tested in three independent experiments on all plates. The plates were incubated at 28 °C for 30 days. Growth was followed by measuring the radial extension of the mycelium. A decolorized zone appeared when the fungus degraded the dye. Daily measurements of the colonies and the decolorized zones (if any) were taken for each strain. Decolorization index (DI) was calculated as the ratio between the decolorized zone and colony diameter. 

### 2.4. Decolorization in Liquid Medium

Decolorization was investigated in a batch system with BRM medium by adding 200 ppm of either the anthraquinonic dye Remazol Brilliant Blue R (RBBR), the azoic Reactive Black 5 (RB5) or the triphenylmethane type Crystal Violet (CV) to 18 day cultures of the evaluated fungi, incubated at 28 °C and 200 rpm. The absorption spectra were measured in the range 200–800 nm at 0, 2, 4, 6, 8 and 24 h. Color reduction was followed spectrophotometrically, and decolorizing activity was calculated from the decrease in absorption of the peak maximum for each dye (592 for RBBR, 597 for RB5, and 588 for CV). Afterwards, the supernatants were collected by filtration and employed for toxicity assays. The dye adsorbed to the mycelium was desorbed with ethanol: water (1:1), and its concentration was calculated in the same way as the percentage of decolorization. For this, the cultures were filtered on Whatman No. 1 paper, and the recovered mycelium was washed with the mentioned dilution of ethanol.

### 2.5. Polyacrylamide Gel Electrophoresis (PAGE) and Activity Staining of Gels

Ligninolytic enzymes and their involvement in dye decolorization were assessed by Sodium Dodecyl Suphate-Polyacrylamide Gel Electrophoresis (SDS-PAGE) of the crude extracts [29]. Separation was carried out on vertical polyacrylamide slab gels. Electrophoresis was performed on 12% polyacrylamide gel under denaturing conditions (SDS-PAGE). The buffer solution for the separating gel was 1.5 mM (pH 8.8). Twenty-five μg of protein culture filtrates from different incubation days were loaded onto the gel and electrophoresed with Tris/Glycine buffer (pH 8.3) at 100 V. Proteins separated in the gel were stained with Coomassie Brilliant Blue R, and their molecular mass was estimated with Precision Plus Protein™ Kaleidoscope™ Prestained Protein Standards (BioRad, Louisville, Kentucky, USA). The zymograms were revealed with DMP as cofactors for LiP (H_2_O_2_) and MnP (H_2_O_2_ and Mn^+2^) activities, respectively. Afterwards, the gel was fixed for 15 min in 10 (v/v) acetic acid and 40 (v/v) methanol, and then soaked in 200 mM acetate buffer (pH 3.5) containing 2 mM/mL of DMP. Protein bands exhibiting laccase activity were stained orange with DMP within 10 min. The anthraquinonic dyes RBBR and Acid Green 27 (AG27) were utilized for the inverse zymograms. The gels were immersed in 200 mM sodium acetate (pH 3.5), with 1 mL 2% AG27 or RBBR. They were shaken gently for 1 h, until evidence of clearing bands appeared. 

### 2.6. Phytotoxicity Bioassay

The phytotoxicity of the dyes and their derivates were determined using *Raphanus sativus* var. Champion and *Pisum sativum* var. Lincoln seeds (IMAISA S.A., Nuevo León, México). They were incubated for 5 days at 25 °C with either (a) control (water); (b) a fresh solution of the dye (200 ppm) or (c) the dye solution treated for 24 h with each fungus. Ten seeds were evaluated in each Petri dish. Every treatment was carried out in triplicate. 

The germination percentage was calculated considering the visible appearance of the radicle [30]. The damage in the radicle (*Rd*) was calculated according to Equation (1):

(1) $$(1):\;Rd=\frac{Radicle\;length\;control_{\;}-Radicle\;length\;test}{Radicle\;length\;control}*100$$

Dye detoxification was estimated using the formula of Sobrero and Ronco [31]:

(2) $$Detoxification=\;\frac{\left(\%T\right)\left(100\right)}{\left(\%C\right)}$$

where %T and %C are respectively, the percentages of inhibition on germination or the damage of the radicle in the treated samples and in the control.

Lethal or sub-lethal damage was determined in the seeds that did not germinate. These seeds were subjected to a test of viability [32]. Seeds that did not germinate were placed in Petri dishes with Whatman paper, and then saturated with 4 mL^−1^ distilled water. The plates were incubated at 25 ± 2 °C, for a period of 4 and 5 days. The amount of seeds that germinated was documented, thus confirming the viability and determining the type of effect the treatments and controls had on the seeds. In those that did not germinate when exposed to the treatments and those in which, when subjected to the simple germination test, the protrusion of the seed by the radicle was observed, sub-lethal damage was considered to have occurred.

### 2.7. Statistical Analysis

Data were subjected to ANOVA using the SPSS Statistics 10.0 (SPSS, Chicago, IL, USA) software program. Data of the analytical determinations are the average of the results of three replications. The significant differences between treatments were compared by Tukey’s test at a 5% level of probability. 

## 3. Results and Discussion

### 3.1. Solid-Plate Dye Decolorization

The broad-spectrum decolorization efficiency of the isolates was evaluated using chemically different dyes representative of the most used dyes (Table 1). Poly R-478, characterized by a chemical complexity similar to lignin compounds, allows the assessment of the fungal capability of degrading lignin and aromatic molecules [33], while Azure B was employed to detect LiP activity [34]. The results obtained are depicted in Table 2. *Trametes* sp. LA1 displayed the best results in Poly R-478 while *T. maxima* LE130 performed best in Azure B (respectively, DI = 0.35 and 0.64 at day 5 of cultivation). Under the conditions assayed, *P. chrysosporium* ATCC 24,725 was reported as an efficient LiP and dye decolorizing strain [35], which only partially decolorized Azure B and Acid Orange (OII) plates after 15 days (Table 2) and Poly R-478 after 30 days (data not shown). No statistically significant differences were registered among *Trametes* sp. LA1 DI of Direct Black (DB), OII, RB5 and RBBR. The best DI values were attained with CV. *T*. *maxima* LE130 rendered the highest DI for all the dyes evaluated. The different DI values reflect the different capacities of the fungal cultures to remove dyes with diverse chemical structures. Small structural differences between the dyes could significantly affect their decolorization. This might be due to differences in electron distribution, charge density or steric factors [36].

### 3.2. Production of Ligninolytic Enzymes in Liquid Media

**Figure 1:**
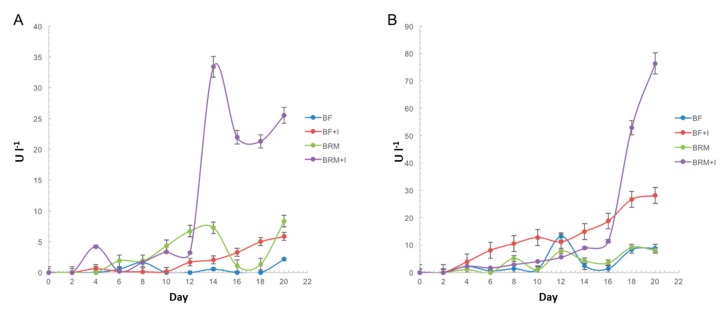
Time course of laccase production in BF or rich medium for Basidiomycota (BRM) media, alternatively supplemented with 0.1 mM MnSO_4_, 0.1 mM FeSO_4_, 0.35 mM CuSO_4_ and 3% ethanol (BF + I, BRF + I) by (**A**) *T. maxima* LE130 and (**B**) *Trametes* sp. LA1. The values are the mean of three replications ± SE.

Among the ligninolytic enzymes evaluated, only laccase activity was detected in the culture fluids of both strains in all the media assayed. The highest titers were quantified in medium BRM supplemented with 0.1 mM MnSO_4_, 0.1 mM FeSO_4_, 0.35 mM CuSO_4_ and 3% ethanol (Figure 1). *Trametes* sp LA1 produced 80 U L^−1^ at day 18, while *T. maxima* LE130 produced 35 U L^−1^ at day 12.

**Figure 2:**
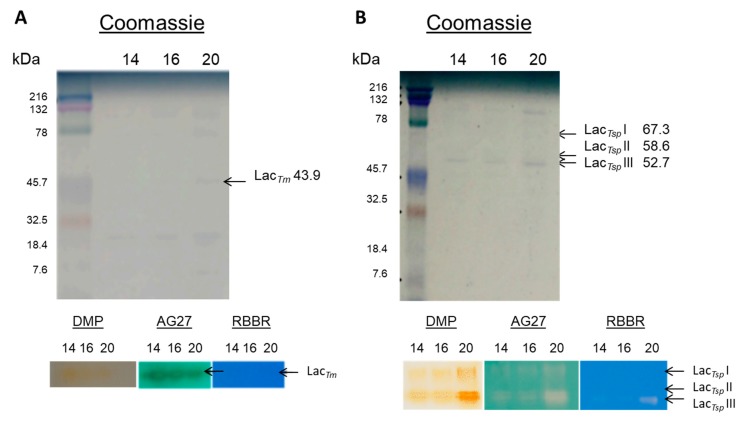
SDS-PAGE and laccase zymograms. (**A**) *T. maxima* LE130 SDS-PAGE and zymograms with 2,6 dimethoxyphenol (DMP), Acid Green 27 (AG27) and RBBR. (**B**) *Trametes* sp. LA1 SDS-PAGE and zymograms with DMP, AG27 and RBBR. Culture supernatants were obtained after 14, 16 and 18 days of incubation.

The absence of noticeable levels of LiP and MnP was confirmed by SDS-PAGE gels, revealed by 2,6-DMP and the cofactors required for each enzyme (H_2_O_2_ and H_2_O_2_ + Mn^+2^, respectively). Laccase was the only activity detected in these gels (Figure 2). In *Trametes* species, laccases are the major enzymes, but peroxidases are also secreted during dye decolorization [7]. Nevertheless, under the conditions assayed in this work, peroxidase activity was not detected. In both strains assayed, the laccase isoenzymatic profile did not vary between days 14 and 20. *T. maxima* LE130 produced a single laccase isoform (Lac*_Tm_*), with a molecular weight of 43.9 kDa. *Trametes* sp LA1 produced three isoforms (Lac*_Tsp_* I, Lac*_Tsp_* II, Lac*_Tsp_* III), with molecular weights of, respectively, 67.3, 58.6 and 52.7 kDa. The molecular masses reported in this study are in the range observed for laccases isolated from other white-rot fungi; usually, molecular masses of fungal laccases range from 30 to 300 kDa [37]. Laccase decolorizing activity was assayed by inverse zymograms, as revealed with the dyes AG27 and RBBR (Figure 2). In *T. maxima* LE130 zymograms stained with AG27 (Figure 2A), a precipitate was observed coincident with the laccase activity band, possibly resulting from dye polymerization. The capacity of phenoloxidases to polymerize phenols is widely documented (for both free and immobilized laccase) [38]. *T. maxima* LE130 laccase activity did not show potential for decolorizing RBBR. RBBR decolorization by *T. maxima* LE130 laccase might require the presence of redox mediators. The potential of synthetic dye decolorization by purified laccases has been described in several species; however, most dyes are only transformed in the presence of redox mediators [39]. Natural laccase mediators [40] may be present in the extracellular fluids of the fungus, which proved capable of decolorizing 55% of this dye after 24 h (Table 3). All laccase isoforms from *Trametes* sp. LA1 showed decolorizing activity on AG27 (Figure 2B), but only Lac*_Tsp_* III was capable of decolorizing RBBR. The isozymes may have different substrate affinity, which may affect their decolorization potential [41]. Finding laccase as the principal activity responsible for decolorization is consistent with previous reports with *Trametes hispida* [42] and *T. hirsuta* [26]. However, further studies with pure enzymes are necessary to corroborate this conclusively.

### 3.3. Decolorization of Dyes by Liquid Cultures

Both strains assayed showed significant differences (*p* ≤ 0.05) in their ability to decolorize dyes with diverse chemical structures. After 24 h, the best results were obtained by *T. maxima* LE130 with the azo dye RB5 (61% decolorization), while the triarylmethane CV was more resistant to biodegradation (Table 3). However, *Trametes* sp. LA1 could decolorize CV more effectively (*p* ≤ 0.05) (approximately 80%). The role of adsorption on the fungal mycelium in dye decolorization was minimal. The dyes were rapidly removed from the medium because of physical adsorption, but they were later eliminated from both the solution and the surface of the mats because of the enzymatic degradation. At the end of the experiment, sorption accounted for less than 5%. Aretxaga et al. [43] as well as Levin et al. [44] obtained similar results when they evaluated dye decolorization by *T. versicolor*. Sorption was responsible for less than 3% of azo and triphenylmethane dye removal by ligninolytic (dye-decolorizing) cultures of *Pycnoporus sanguineus* [45].

### 3.4. Phytotoxicity Assays

The phytotoxicity of CV, RBBR and RB5 before and after treatment with *T.maxima* LE130 and *Trametes* sp. LA1 cultures was assessed by the analysis of their effects on seed germination, radicle length and damage, using *R. sativus* (Table 4) and *P. sativum* seeds (Table 5). CV was the only dye which altered *R. sativus* germination. However, all the dyes assayed caused severe damages to the radicle (approximately 90%). Treated dyes with *T. maxima* LE130 were more toxic than the untreated ones (showing significant differences not only in seed germination percentages but also in radicle length). Treatment with *Trametes* sp. LA1 reduced the toxicity of the triphenylmethane dye CV; treated dyes did not affect the germination of these seeds, but also markedly diminished radicle damage (55% decrease) and growth inhibition in comparison with the untreated dyes. Laccase activity might be involved in decolorization and detoxification processes, considering that *Trametes* sp. LA1 laccase titers in cultures applied in decolorization (80 U L^−1^) were more than twice those registered in *T. maxima* LE130 culture fluids, and concomitantly, *Trametes* sp. LA1 was capable of decolorizing approximately 80% of 200 ppm CV, while *T. maxima* LE130 cultures only decolorized 40% of this dye (Figure 1, Table 3). Germination percentages did not vary significantly between RB5-untreated and *Trametes* sp. LA1-treated samples, but radicle damage decreased (8%) and length increased with *Trametes* sp. LA1 treatment. On the contrary, *Trametes* sp. LA1-treated RBBR slightly inhibited growth germination and decreased root elongation. The effects varied when assaying *P. sativum* seeds, but CV was the only dye that affected both radicle length and damage. Treated dyes with *T. maxima* LE130 were more toxic than the untreated ones (the root lengths of selected plants decreased with all the treated dyes and germination diminished when applying *T. maxima* LE130-treated CV). However, CV metabolites present after with *Trametes* sp. LA1-treatment did not affect seed germination, although the negative effects of CV on root elongation and damage persisted. *P. sativum* root elongation was neither affected by RBBR and RB5 nor by the metabolites which resulted after treatment. Radicle damage was also not observed. The abiotic controls as well as the three dyes treated with *T. maxima* LE130 and *Trametes* sp. LA1-treated RB5 and RBBR only produced sub-lethal effects on both seeds, as they conserved their germination capacity.

**Figure 3:**
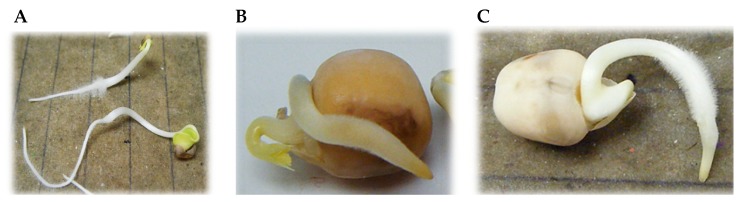
(**A**) Damage observed in *R. sativus* seedlings exposed 5 days to RB5 treated *T. maxima* LE130 cultures; (**B**) *P. sativum* seedlings after being exposed 5 days to the LE130-treated dye; (**C**) *P. sativum* seedling exposed to CV treated with *Trametes* sp. LA1 (LA1-treated dye).

Damage was registered in *R. sativus* cotyledons as a result of dye exposition (Figure 3A) and in radicle calyptra of *P. sativum* seeds (Figure 3B). Treatment with *Trametes* LA1 diminished the damage observed (Figure 3C).

In overview, the phytotoxicity of the azoic dye RB5 and the triphenylmethane CV treated with *Trametes* sp. LA1 decreased by around 15% and 45% when tested in *R. sativus* seeds, while the toxicity of CV and the anthraquinonic dye RBBR diminished by approximately 42% and 48% when assayed with *P. sativum* seeds (Table 6). On the contrary, *T. maxima* LE130-treated dyes were more toxic than the abiotic controls. RB5 toxicity decreased after treatment with *Phanerochaete sordida* laccase [46]. The laccase from *Trametes pubescens* also decreased the toxicity of the azo dye Congo Red [47] (Si et al., 2013). The degradation of azo dyes OII and Acid Orange 6 by laccase from *T. versicolor* was also accompanied by a decrease of phytotoxicit [10]. The triphenylmethane dye Malachite Green proved to be detoxified by *Pleurotus florida* [48] and *Fomes sclerodermeus* [49] laccases. However, the results may vary with the dye assayed; for example, Ramsay and Nguyen (2002) [14] treated various textile dyes with *T. versicolor*. After decolorization, the toxicity of the solutions containing Amaranth, Tropaeolin O and RB5 was unchanged and Reactive Blue 15, RBBR and Cibacron Brilliant Red 3G-P decreased to non-toxic levels, but Cibacron Brilliant Yellow 3B-A and Congo Red became very toxic. 

## 4. Conclusions

There is currently growing interest in assessing the potential of new microorganisms for industrial and environmental applications, including wastewater treatment. More than 70% of freshwater resources in Mexico are affected as a result of pollution from all sources, with 31% described as contaminated or grossly contaminated [50]. *Trametes* sp. LA1—a new, isolated strain from Mexico— when cultivated in a low-cost medium based on bran flakes, produced laccase as the main ligninolytic enzyme detected in its culture fluids and was capable of decolorizing and detoxifying chemically diverse dyes, without the addition of redox mediators. The major drawback of the enzymatic treatment is the inactivation of enzymes in the conditions normally found in textile wastewater and the high cost of enzyme production [51]. Based on these results, it is suggested that *Trametes* sp. LA1 might be a notable candidate for wastewater biodecolorization.

## Figures and Tables

**Table 1:** Dyes used in the experiment, their commercial and color index (CI) number, acronym, class and chemical structure.

Commercial Name	CI Number	Acronym	Dye Class	Chemical Structure
Basic Violet 3 (Crystal Violet)	42555	CV	Triphenyl methane	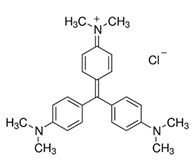
Direct Black 22 (Direct Black CA)	35435	DB22	Azo	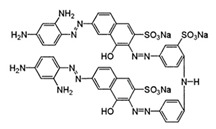
Acid Orange 7 (Orange II)	15510	OII	Azo	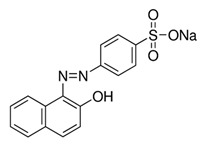
Reactive Black 5 (Remazol Black B)	20505	RB5	Azo	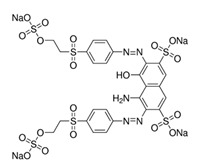
Reactive Blue 19 (Remazol Brilliant Blue R)	61200	RBBR	Anthraquinone	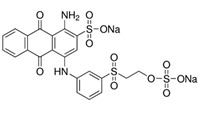
(Methylene Azure B)	52010	AB	Heterocyclic	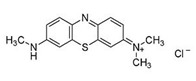
Poly R-478		PR	Polymeric	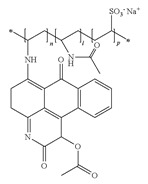

**Table 2:** Solid-plate dye decolorization in Bran Flakes (BF) medium supplemented with 200 ppm of each of the dyes evaluated.

Strain/ Days	Decolorization Index (DI)							SE/*p*-Value
	PR	AB	CV	DB22	OII	RB5	RBBR	
**A**								0.016/>0.05
5	0.000	0.000	0.000	0.000	0.000	0.000	0.000	
7	0.000	0.000	0.000	0.000	0.000	0.000	0.000	
15	0.000	0.063	0.000	0.000	0.042	0.000	0.000	
**B**								0.121/0.0001
5	0.00b	0.639a	0.000b	0.000b	0.690a	0.866a	0.778a	
7	0.000b	0.847a	0.000b	0.000b	0.822a	0.908a	0.932a	
15	1.000a	1.000a	2.543a	1.000a	1.000a	1.000a	1.000a	
**C**								0.278/>0.05
5	0.350	0.316	0.000	0.282	0.363	0.364	0.358	
7	0.369	0.361	1.333	0.369	0.358	0.381	0.393	
15	0.413	0.413	1.059	0.413	0.413	0.413	0.413	

(A) *P. chrysosporium* ATCC 24725, (B) *T. maxima* LE130, (C) *Trametes* sp. LA1. SE: standard error of the mean. ^a–b^ Means in rows with different superscripts indicate significant differences between treatments (*p* ≤ 0.05).

**Table 3:** Decolorization of dyes by liquid cultures of *T. maxima* LE130 and *Trametes* sp. LA1 grown in BRM medium at 28 °C. Decolorization was investigated by adding 200 ppm of either the anthraquinonic dye RBBR, the azoic RB5 or the triphenylmethane CV to 18 day cultures of the evaluated fungi.

T. Maxima LE130					
Time (h)	Decolorization (%)			Parameter	*p*-value
	CV	RB5	RBBR		
0	0.00	0.00	0.00	Model	0.0312
2	12.95	43.92	45.23	Dye (D)	0.0003
4	25.40	51.50	46.17	Time (t)	0.1875
6	27.58	51.66	46.54	D * t	0.9884
8	27.47	55.19	47.74		
24	41.05	61.01	54.94		
SE (t)	7.84	7.84	8.49		
C (mean ± SD)	26.89 ± 3.5 ^b^	52.66 ± 3.5 ^a^	48.12 ± 3.84 ^a^		
Trametes sp. LA1					
0	0.00	0.00	0.00	Model	0.0028
2	63.40	44.45	39.50	Dye (D)	≤0.0001
4	70.80	50.00	44.90	Time (t)	0.3103
6	74.45	51.00	43.70	D * t	0.9946
8	76.60	46.90	42.90		
24	79.70	54.80	49.60		
SE (t)	6.17	6.17	8.72		
C (mean ± SD)	72.99 ± 2.76 ^a^	49.43 ± 2.76 ^b^	44.12 ± 3.02 ^b^		

C: mean and standard deviation (SD) by columns. ^a–b^ Means in rows with different superscripts indicate significant differences between treatments (*p* ≤ 0.05). SE: standard error of the mean.

**Table 4:** Phytotoxicity of the dyes on *R. sativus* seeds before and after 24 h treatment with *T. maxima* LE130 and *Trametes* sp. LA1 cultures.

Dye ^Ψ^	Control *	Untreated Dye	LE130-Treated Dye	LA1-Treated Dye	SE/*p*-Value
**Germination inhibition (%)**					5.53/≤0.0001
CV	3.33 ^c; A^	56.67 ^b; A^	83.33 ^a; A^	0.00 ^c; B^	
RB5	3.33 ^b; A^	16.67 ^b; B^	40.00 ^a; B^	16.67 ^b; A^	
RBBR	3.33^b; A^	6.67 ^b; B^	93.33 ^a; A^	20.00 ^b; A^	
**Radicle length (cm)**					0.08/≤0.0001
CV	5.87 ^a; A^	0.21 ^c; B^	0.05 ^c; A^	2.71 ^b; A^	
RB5	5.87 ^a; A^	0.72 ^c; A^	0.26 ^d; A^	1.71 ^b; B^	
RBBR	5.87 ^a; A^	0.43 ^b; B^	0.18 ^c; A^	0.40 ^b; C^	
**Radicle damage (%)**					1.37/≤0.0001
CV	0.00 ^d; A^	96.36 ^a; A^	99.15 ^a; A^	53.82 ^b; C^	
RB5	0.00 ^d; A^	87.80 ^b; B^	95.64 ^a; A^	70.85 ^c; B^	
RBBR	0.00 ^c; A^	92.70 ^b; A^	96.88 ^a; A^	93.12 ^a b; A^	

**^Ψ^** All tests were performed with 2 mg mL^−1^ at 25 °C, which is a concentration below the water-solubility limit of each of the dyes (50, 550 and 10–50 mg ml^−1^ for CV, RB5 RBBR, respectively). * Control: seeds treated with water. SE: standard error of the mean. ^a–d^ Means in rows with different superscripts indicate significant differences between treatments (*p* ≤ 0.05). ^A–C^ Means in columns with different superscripts indicate significant differences between dyes (*p* ≤ 0.05).

**Table 5:** Phytotoxicity of the dyes on *P. sativum* seeds before and after 24 h treatment with *T. maxima* LE130 and *Trametes* sp. LA1 cultures.

Dye ^Ψ^	Control *	Untreated Dye	LE130-Treated Dye	LA1-Treated Dye	SE/*p*-Value
**Germination inhibition (%)**					3.97/≤0.0001
CV	6.67 ^b; A^	6.67 ^b; A^	50.00 ^a; A^	0.00 ^b; A^	
RB5	6.67 ^a; A^	0.00 ^a; A^	0.00 ^a; B^	0.00 ^a; A^	
RBBR	6.67 ^a; A^	0.00 ^a; A^	3.33 ^a; B^	0.00 ^a; A^	
**Radicle length (cm)**					0.05/≤0.0001
CV	1.27 ^a; A^	0.75 ^b; B^	0.49 ^c; A^	0.82 ^b; B^	
RB5	1.27 ^a; A^	1.49 ^a; A^	0.67 ^c; A^	1.12 ^a b; A^	
RBBR	1.27 ^a; A^	1.32 ^a; A^	0.58 ^b; A^	1.36 ^a; A^	
**Radicle damage (%)**					3.33/≤0.0001
CV	0.00 ^c; A^	40.67 ^b; A^	61.33 ^a; A^	35.33 ^b; A^	
RB5	0.00 ^b; A^	0.00 ^b; B^	47.67 ^a; A^	14.67 ^b; B^	
RBBR	0.00 ^b; A^	2.67 ^b; B^	54.67 ^a; A^	0.00 ^b; B^	

**^Ψ^** All tests were performed with 2 mg mL^−1^ at 25 °C, concentration below the water-solubility limit of each of the dyes (50, 550 and 10–50 mg mL^−1^ for CV, RB5 RBBR, respectively). * Control: seeds treated with water. SE: standard error of the mean. ^a–b^ Means in rows with different superscripts indicate significant differences between treatments (*p* ≤ 0.05). ^A–B^ Means in columns with different superscripts indicate significant differences between dyes (*p* ≤ 0.05).

**Table 6:** Detoxification determined with *R. sativus* or *P. sativum* seeds.

Seed	RB5		CV Detoxification (%)		RBBR	
	LE130	LA1	LE130	LA1	LE130	LA1
*R. sativus*	0 ^a^	15.24 ^a^	0 ^a^	44.39 ^a^	0 ^a^	0 ^b^
*P. sativum*	0 ^a^	0 ^b^	0 ^a^	41.71 ^b^	0 ^a^	48 ^a^

^a–b^ Means in columns with different superscripts indicate significant differences between treatments (*p* ≤ 0.05).
